# Piebaldisme: une génodermatose rare

**DOI:** 10.11604/pamj.2017.27.221.4961

**Published:** 2017-07-24

**Authors:** Fatima Zahra Debbarh, Fatima Zahra Mernissi

**Affiliations:** 1 Service de Dermatologie, CHU Hassan II, Fès, Maroc

**Keywords:** Piebaldisme, génodermatose, c-kit, macules achromiques, Piebaldism, génodermatosis, c-kit, achromic macules

## Abstract

Piebaldism is a rare autosomal dominant genodermatosis. It is due to congenital absence of melanocytes in the affected areas. We report a case. A 5 year old girl born to consanguineous parents and with similar cases in her mother’s; she presented since birth achromic lesions on the legs with a steady evolution. clinical examination showed confluent achromic macules and poliosis (A) with no contrast enhancement under Wood lamp and several coffee-with-milk colored spots on the trunk and thighs(B). The diagnosis of piebaldism was made. Piebaldism is a rare genodermatosis. Its incidence is estimated at less than 1/20000 newborns. It is characterized by the congenital absence of melanocytes in the areas affected by mutation of the c-kit gene and by symmetrical achromic macules appeared at birth with a steady and persistent evolution. A white lock of hair on the forehead could be seen in 80% of cases The differential diagnosis includes vitiligo, albinism and Waardenburg syndrome. Associations have been described with neurofibromatosis type I. However, isolated coffee-with-milk colored spots can be observed; as the case of our patient. The treatment is based on split-thickness skin graft. Piebaldism is a rare genodermatosis. This study aims to discuss its clinical aspects and differential diagnoses.

## Image en médecine

Le piebaldisme est une génodermatose rare de transmission autosomique dominante. Il est du à l'absence congénitale des mélanocytes aux zones touchées. Nous en rapportant un cas. Une fille de 5 ans; avec notion de consanguinité et cas similaire chez la mère; a présenté dès la naissance des lésions achromiques des jambes d'évolution stable. L'examen a objectivé des nappes achromiques des jambes avec ilots de pigmentation et poliose (A); sans accentuation du contraste à la lumière de Wood et multiples tâches café au lait au niveau du tronc et cuisses (B). Le diagnostic de piebaldisme a été posé. Le piebaldisme est une génodermatose rare. Son incidence est estimée à moins de 1/20000 naissance. Il est caractérisé par l'absence congénitale de mélanocytes aux zones touchées par mutation du gène c-kit et se manifeste par des macules achromiques symétriques dès la naissance d'évolution stable et persistante. Une mèche de cheveux blanche au niveau frontal pourrait se voire dans 80% des cas. Le diagnostic différentiel se fait avec le vitiligo; l'albinisme et le syndrome de Waardenburg. Des associations ont été décrites avec la neurofibromatose de type I. Cependant; des tâches café au lait isolées peuvent être observé; comme le cas de notre patiente. Le traitement est basé sur la greffe de peau mince. Le piebaldisme est une génodermatose rare. A travers cette observation; on discute ses aspects cliniques et ses diagnostics différentiels.

**Figure 1:**
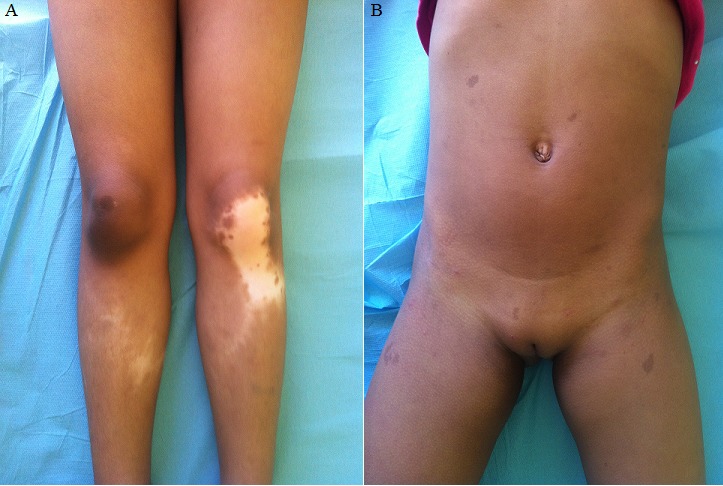
A) macules achromiques des jambes confluentes; B) multiples tâches café au lait du tronc et cuisses

